# Novel artificial nerve transplantation of human iPSC-derived neurite bundles enhanced nerve regeneration after peripheral nerve injury

**DOI:** 10.1186/s41232-024-00319-4

**Published:** 2024-02-13

**Authors:** Takayuki Nishijima, Kentaro Okuyama, Shinsuke Shibata, Hiroo Kimura, Munehisa Shinozaki, Takehito Ouchi, Yo Mabuchi, Tatsukuni Ohno, Junpei Nakayama, Manabu Hayatsu, Keiko Uchiyama, Tomoko Shindo, Eri Niiyama, Sayaka Toita, Jiro Kawada, Takuji Iwamoto, Masaya Nakamura, Hideyuki Okano, Narihito Nagoshi

**Affiliations:** 1 Department of Orthopaedic Surgery, Keio University School of Medicine, 35 Shinanomachi Shinjuku-Ku, Tokyo, 160-8582 Japan; 2 Department of Physiology, Keio University School of Medicine, 35 Shinanomachi Shinjuku-Ku, Tokyo, 160-8582 Japan; 3 Division of Microscopic Anatomy, Niigata University Graduate School of Medical and Dental Sciences, 1-757 Asahimachi-Dori, Chuo-Ku, Niigata, 951-8510 Japan; 4 Electron Microscope Laboratory, Keio University School of Medicine, 35 Shinanomachi Shinjuku-Ku, Tokyo, 160-8582 Japan; 5 Department of Orthopaedic Surgery, Kitasato Institute Hospital, 9-1, Shirokane 5-Chome, Minato-Ku, Tokyo, 108-8642 Japan; 6 Department of Physiology, Tokyo Dental College, 2-9-18, Kanda-Misaki-Cho, Chiyoda-Ku, Tokyo, 101-0061 Japan; 7 Department of Clinical Regenerative Medicine, Fujita Medical Innovation Center, Fujita Health University, Floor 4, Haneda Innovation City Zone A, 1-1-4, Hanedakuko, Ota-Ku, Tokyo, 144-0041 Japan; 8 Oral Health Science Center, Tokyo Dental College, 2-9-18 Kanda-Misaki-Cho, Chiyoda-Ku, Tokyo, 101-0061 Japan; 9 Jiksak Bioengineering, Inc, Cybernics Medical Innovation Base-A Room 322, 3-25-16 Tonomachi, Kawasaki-Ku, Kawasaki-Shi, Kanagawa, 210-0821 Japan; 10 Present address: Faculty of Materials for Energy, Graduate School of Natural Science and Technology, Shimane University, Matsue, Shimane Japan

**Keywords:** Peripheral nerve injury, Artificial nerve, Neurite bundle, hiPSC

## Abstract

**Background:**

Severe peripheral nerve damage always requires surgical treatment. Autologous nerve transplantation is a standard treatment, but it is not sufficient due to length limitations and extended surgical time. Even with the available artificial nerves, there is still large room for improvement in their therapeutic effects. Novel treatments for peripheral nerve injury are greatly expected.

**Methods:**

Using a specialized microfluidic device, we generated artificial neurite bundles from human iPSC-derived motor and sensory nerve organoids. We developed a new technology to isolate cell-free neurite bundles from spheroids. Transplantation therapy was carried out for large nerve defects in rat sciatic nerve with novel artificial nerve conduit filled with lineally assembled sets of human neurite bundles. Quantitative comparisons were performed over time to search for the artificial nerve with the therapeutic effect, evaluating the recovery of motor and sensory functions and histological regeneration. In addition, a multidimensional unbiased gene expression profiling was carried out by using next-generation sequencing.

**Result:**

After transplantation, the neurite bundle-derived artificial nerves exerted significant therapeutic effects, both functionally and histologically. Remarkably, therapeutic efficacy was achieved without immunosuppression, even in xenotransplantation. Transplanted neurite bundles fully dissolved after several weeks, with no tumor formation or cell proliferation, confirming their biosafety. Posttransplant gene expression analysis highlighted the immune system’s role in recovery.

**Conclusion:**

The combination of newly developed microfluidic devices and iPSC technology enables the preparation of artificial nerves from organoid-derived neurite bundles in advance for future treatment of peripheral nerve injury patients. A promising, safe, and effective peripheral nerve treatment is now ready for clinical application.

**Supplementary Information:**

The online version contains supplementary material available at 10.1186/s41232-024-00319-4.

## Background

Most severe peripheral nerve injuries do not heal without surgical intervention [1, 2]. End-to-end nerve suturing is only adoptable for short gaps, while nerve transplantation is needed for large defects. Currently, autologous nerve grafts are the gold standard technique [1–4]. However, harvesting autografts has several drawbacks, such as painful neuroma formation and graft length limitation [5, 6]. Other options, such as allogeneic nerve or artificial nerve transplantation, do not produce sufficient outcomes; thus, various studies have been conducted, many of them focusing on generating novel effective nerve conduits for enhancing nerve regeneration [7–10].

Here, we generated novel artificial nerves by utilizing a special culture technology that separately cultures cell bodies and neurite bundles of nerve organoids differentiated from human induced pluripotent stem cells (hiPSCs) [11–13]. The special culture microfluidic device contains chambers with microslits for receiving spheroids and forming straight and unidirectional neurites, which enable the separation of organoid cell bodies and neurites, resulting in neurite bundles that can be used to study various diseases [14, 15]. Compared to random directional fibers, aligned polymer fibers were reported to promote in vivo nerve regeneration, suggesting that straight and unidirectional parallel neurite bundle bridging is suitable as a scaffold [16]. After parallelly aligning the cell-free neurite bundles, a novel artificial nerve conduit for transplantation was created by assembling several neurite bundles from various subtypes of neurons, similar to an acellularized allogeneic nerve graft.

Since hiPSC-derived neuronal spheroids are transferred into custom-made microdevices, the neurite bundle length, diameter, and subtypes of neurons can be selected by modifying the device structure and the procedure of hiPSC differentiation. Previous reports demonstrated that compared to that of sensory nerve grafts, which are normally used for clinical autologous nerve grafting, transplantation of motor nerve grafts increased nerve regeneration after injury [17–19]. By using various subtypes of nerve organoids, it is possible to evaluate the effectiveness of various neuronal subtype neurite bundles, which are most beneficial for peripheral nerve regeneration.

In this study, a quantitative comparison was performed over time to search for the artificial nerve with the greatest therapeutic effect, evaluating the recovery of motor and sensory functions and histological regeneration. In addition, to elucidate the mechanism of promoting nerve regeneration in the group with high therapeutic effects, a multidimensional unbiased evaluation was carried out by analyzing the expression profiling using next-generation sequencing.

## Results

### Transplantation of newly generated artificial nerves using a microfluidic device

**Fig. 1:**
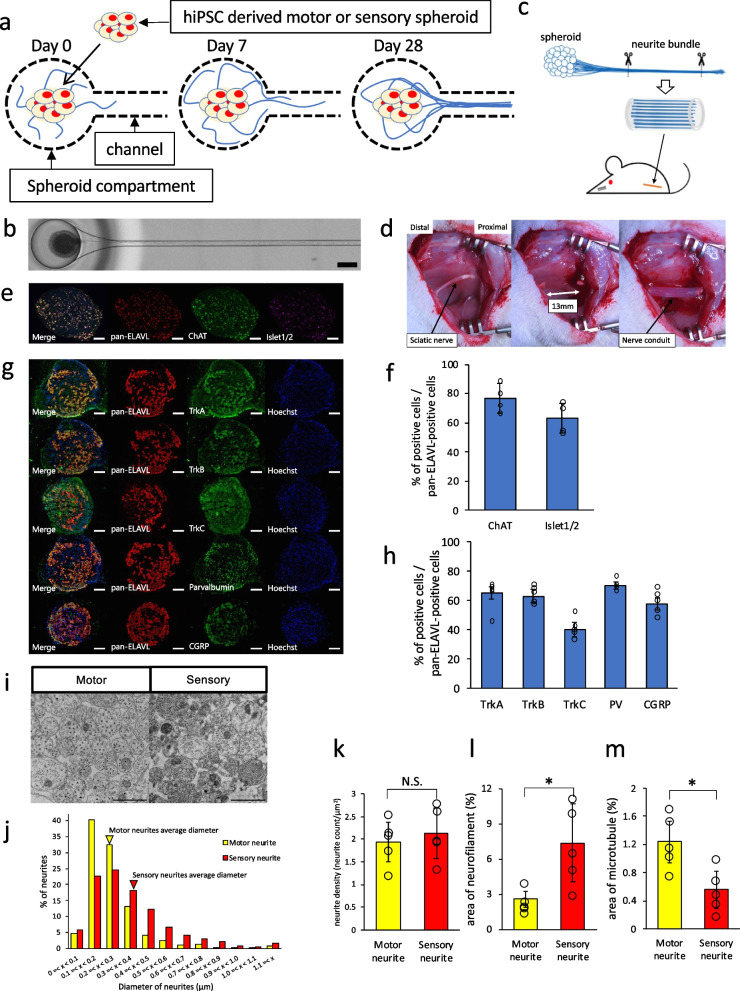
Generation and implantation procedure of novel artificial nerves with motor and sensory neurite bundles. **a** Schematic diagram of creating nerve organoids with neurite bundles derived from spheroids. **b** Whole image of the nerve organoid cultured in the microfluidic device. Scale bar = 300 μm. **c**, Schematic explanation of the procedure for neurite bundle-derived artificial nerve creation by removing spheroids that contained cell bodies from the nerve organoids. **d** The implantation procedure of a nerve conduit tube at the transected sciatic nerve with a 13 mm gap. **e** Immunofluorescence images of ChAT and Islet1/2 staining of motor neuron spheroids derived from hiPSCs. Scale bars = 100 μm. **f** Quantitative analysis of ChAT- and Islet1/2-positive cells in pan-ELAVL-positive cells. **g** Immunofluorescence images of TrkA, TrkB, TrkC, PV (parvalbumin) and CGRP staining of sensory neuron spheroids derived from hiPSCs. Scale bars = 100 μm. **h** Quantitative results of TrkA-, TrkB-, TrkC-, PV (parvalbumin)- and CGRP-positive cells in pan-ELAVL-positive cells. **i**, Electron microscopic images of the axial section from motor and sensory neurite bundles consisting of condensed neural fibers. Scale bars = 1 μm. **j** Neurite diameter distribution in motor and sensory neurite bundles. Arrowheads indicate the position of the average diameter. **k** Average neurite density in neurite bundles. **l**,** m** Average ratio of neurofilaments’ area (**l**) and microtubules’ area (**m**) in neurite area. (*n* = 5) *p < 0.05, N.S. = not significant. Data are represented as the mean ± standard error of the mean (SEM)

To create neurite bundle-derived artificial nerves, a microfluidic device was used to culture spheroid cell bodies and neurites separately (Fig. 1a and Supplementary Figure 1a). After the motor and sensory neuronal spheroids were placed in the spheroid compartment and cultured for approximately 4 weeks, a neurite bundle of approximately 2 cm in length was formed in the channel. (Fig. 1a, b). Electron microscopic examination of the axial neurite bundles revealed a dense concentration of unmyelinated neurites (Supplementary Figure 1b). A single neurite bundle contains approximately several thousand unmyelinated neurites. Spheroids were dissected from the nerve organoids, and six neurite bundles were longitudinally placed in a 15-mm-long silicone tube with collagen gel to create novel artificial nerves for transplantation (Fig. 1c). The conduits with and without neurite bundles were implanted in the transected sciatic nerve in rats, and their effectiveness was evaluated (Fig. 1d).

### Evaluation of hiPSC-derived motor and sensory spheroids and their neurites

Since it is possible to generate motor and sensory neurons from hiPSCs, we decided to transplant motor and sensory neurite bundles into rats and named these groups “motor TP” and “sensory TP”, respectively. To confirm the characteristics of motor and sensory neuronal spheroids, frozen spheroids sections were evaluated by immunostaining. The motor neuron spheroids were stained with ChAT and Islet1/2 to confirm the characteristics of the motor neuron markers (Fig. 1e). The ratio of ChAT-positive in pan-ELAVL-positive cells was 76.4 ± 10.1% and that of Islet1/2-positive in pan-ELAVL-positive cells was 63.3 ± 10.2% (Fig. 1f). We confirmed that the majority of neurons in motor spheroids expressed the mature motor neuron marker ChAT. To evaluate the sensory neuronal characteristics, the sensory spheroids were immunostained with TrkA (tropomyosin receptor kinase A), TrkB (tropomyosin receptor kinase B), TrkC (tropomyosin receptor kinase C), PV (parvalbumin), and CGRP (calcitonin gene-related peptide), (Fig. 1g). The ratio of each sensory neuronal marker-positive cell in the pan-ELAVL-positive cells was 65.3 ± 3.9% for TrkA, 62.8 ± 4.7% for TrkB, 40.2 ± 5.2% for TrkC, 70.5 ± 2.5% for PV and 57.5 ± 4.0% for CGRP (Fig. 1h). We confirmed that several sensory neuron markers responsible for tactile sensation were expressed in sensory spheroids originating from hiPSCs. These results indicated that the neurite bundles from spheroids with motor or sensory nerve properties were successfully prepared from hiPSCs.

Next, we examined the motor and sensory neurite bundles histologically (Fig. 1i). The variation of the neurite diameter in the bundles showed that the diameter of sensory neurites was much larger than that of motor neurites (Fig. 1j). There was no difference in the density of neurites between the motor and sensory groups (Fig. 1k). The detailed comparison of the microstructure inside the neurites by EM revealed that neurofilaments dominantly occupy a larger area in sensory neurite bundles, whereas microtubules occupy a larger area in motor neurites (Fig. 1l and m). We then decided to transplant these motor and sensory neurite bundles, which have different gene expression and also have different neurite bundle properties, into nerve defects as grafts.

### Accelerated regeneration after transplantation

**Fig. 2:**
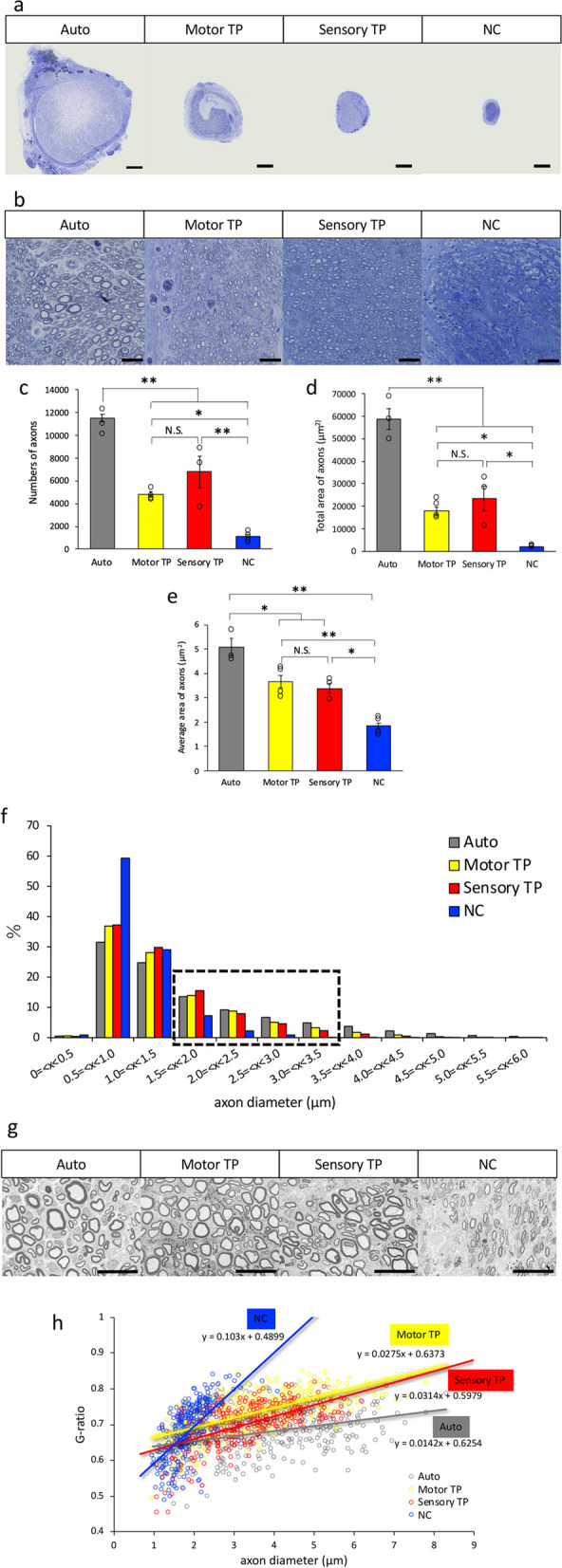
Light and electron microscopy images and quantitative analysis of regenerating axons and myelin sheaths. **a**, **b**  Representative low-magnification full images (a) and high-magnification images (b) of an axial section in the central part of the regenerating nerve, Scale bars = 200 μm (a) and 40 μm (b). **c-e** Quantitative analysis of axon number (c), total axonal area (d), and average area per axon (e), with significant differences between the TP and NC groups. (*n *= 6) **f**  There was variation in the axonal diameters of regenerating nerves, and larger diameter axons (black dotted square area) dominantly recovered in the TP group and Auto group. **g**  Representative electron microscopy images of axial sections in the central part of the regenerating sciatic nerve in each group. Scale bar = 20 μm. **h**  Quantitative analysis of myelin thickness with G-ratio analysis. Both the motor and sensory TP groups showed thick myelination similar to that in the Auto group. (*n *= 5 in Auto, *n* = 5 in Motor TP, *n* = 4 in Sensory TP, *n* = 7 in NC) * *p *< 0.05, ** *p *< 0.01, N.S. = not significant. Data are represented as the mean ± SEM

To evaluate the degree of regeneration, in vivo histological recovery posttransplantation was assessed at 12 weeks. The sciatic nerves from each experimental group were harvested and fixed and then examined using an optical microscope. In axial toluidine blue-stained sections from the center of the regenerated nerve, the TP group exhibited significantly larger numbers and larger areas of regenerated nerve fibers than the NC group, indicating the enhanced recovery of numerous axons (Fig. 2a, b). Quantitative analysis of the axonal count and total axonal area was conducted (Fig. 2c-e). Both the motor TP and sensory TP groups consistently manifested favorable transplantation effects, similar to the Auto group. In every quantification, significant therapeutic effects were evident in the analysis of axon count (Fig. 2c), total axon area (Fig. 2d), and the average area per axon (Fig. 2e) in the treatment groups compared to the NC group (Number of axons: Auto = 11,546 ± 342, Motor TP = 4839 ± 206, Sensory TP = 6781 ± 1400, NC = 1163 ± 142. Total area of axons: Auto = 58,870 ± 4560 μm^2^, Motor TP = 17,888 ± 1888 μm^2^, Sensory TP = 23,500 ± 5429 μm^2^, NC = 2123 ± 190 μm^2^. The average area of axons: Auto = 5.09 ± 0.35 μm^2^, Motor TP = 3.66 ± 0.26 μm^2^, Sensory TP = 3.38 ± 0.20 μm^2^, NC = 1.86 ± 0.11 μm^2^.).

To further assess the detailed effect, the variation in the thickness of regenerated nerves was quantified. The NC group had 10.55% of the regenerated axons located in the range of thicker nerves with diameters ranging from 1.5 to 3.5 μm (black dotted square area), whereas the Auto group had 34.24%, the motor TP group had 31.13%, and the sensory TP group had 30.43% (Fig. 2f). The diameter distribution of regenerating axons in the TP group resembled that in the Auto group, while that in the NC group was limited. These results indicated that the transplantation of novel artificial nerves resulted in the greater regeneration of thicker axons.

### Acceleration of thick myelination observed with electron microscopy

To further evaluate histologically, electron microscopy analysis of the sciatic nerve was conducted at 12 weeks postsurgery. Numerous myelinated regenerated axons were observed in both the Auto group and the TP group (Fig. 2g). The G-ratio, was plotted along with the axon thickness (X-axis) and graphically presented with an approximate linear line (Approximate linear equations, Auto; y = 0.0142x + 0.6254, Motor TP; y = 0.0275x + 0.6373, Sensory TP; y = 0.0314x + 0.5979, NC; y = 0.103x + 0.4899.) (Fig. 2h). The standard treatment Auto group indicated the presence of considerably thicker axons with thicker myelin sheaths, while the NC group indicated the presence of very thin myelin surrounding regenerated axons with a smaller diameter. The straight lines of the motor and sensory TP groups were close to the Auto group, suggesting comparable myelin thickness. These results suggested that transplantation of neurite bundle-derived artificial nerves may promote myelination.

### Functional recovery of lower extremity movement and sensation with transplantation

**Fig. 3:**
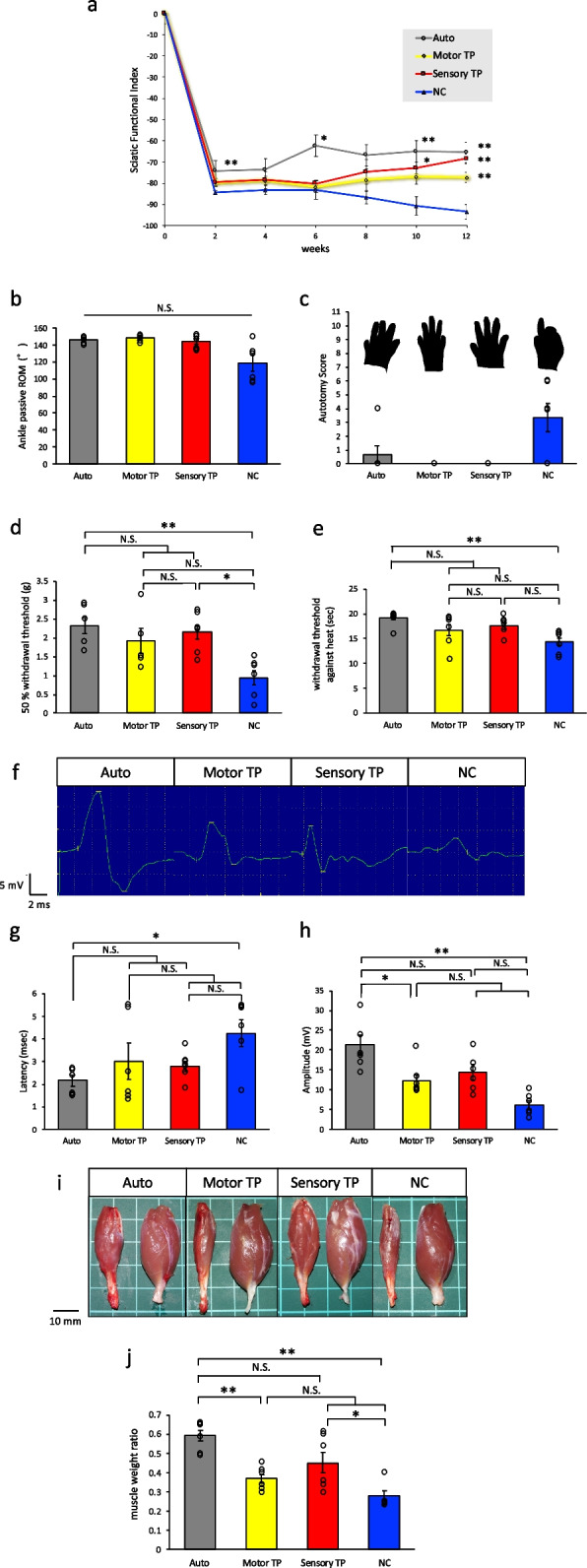
Assessment of motor and sensory functional recovery. **a** Sciatic functional index measured over time after transplantation. Compared to the NC group, the Auto group and TP group were significantly recovered at 12 weeks after transplantation. **b** Ankle joint ROM measurements collected while the animals were under anesthesia demonstrated decreased values only in the NC group. **c** Injury status of toes was measured as the autotomy score. The black shadow shows the contour of the rat’s foot at the time of observation. **d**, **e** Results from the von Frey monofilament test for touch sensation and from the Hargreaves plantar test for thermal sensation demonstrated that the recovery in the motor and sensory TP group was greater than that in the NC group. **f**–**h** From the captured raw images of the waves (f), latency and amplitude were analyzed by measuring the compound muscle action potentials, with improved tendency in TP groups. **i**, **j** The ratio of gastrocnemius wet weight between the injured side and healthy side showed the recovery status of innervated muscles. A significant difference between the sensory TP group and the NC group was detected. Left = injured side. Right = control healthy side. Scale bar = 10 mm (*n* = 6) * *p* < 0.05, ** *p* < 0.01, N.S. = not significant. Data are represented as the mean ± SEM

To evaluate the functional recovery, gait tracking analysis was performed to calculate the sciatic functional index (SFI), (Fig. 3a). Compared to the NC group, the Auto group, the sensory TP group and the motor TP group showed better functional recovery over time. At 12 weeks posttransplantation, the SFI of the Auto group and those of the motor and sensory TP groups were significantly higher than that of the NC group (Auto =  − 65.5 ± 2.1, Motor TP =  − 77.3 ± 2.6, Sensory TP =  − 68.4 ± 2.1, NC =  − 93.4 ± 3.4.). Ankle passive range of motion (ROM) was also measured to assess ankle joint contracture (Auto = 146.3 ± 1.7°, Motor TP = 148.5 ± 2.1°, Sensory TP = 144.0 ± 3.5°, NC = 118.7 ± 9.1°.) (Fig. 3b). The ROMs in the Auto and TP groups were preserved, and no ankle contractures were observed; in contrast, the ROMs of some samples in the NC group showed some limitations (Supplementary Figure 2). These results indicated that transplantation of novel artificial nerves improved motor function.

It is known that rats injure their own toes when their sensitivity is reduced, and the degree of unusual sensitivity is assessed using an autotomy score. The autotomy scores of the motor and sensory TP groups at 12 weeks after transplantation were always zero because self-injury was never detected, suggesting limited reduced sensitivity compared to the Auto and NC groups (Auto = 0.67 ± 0.6, Motor TP = 0 ± 0, Sensory TP = 0 ± 0, NC = 3.3 ± 1.0.) (Fig. 3c). The von Frey monofilament test was used to evaluate touch sensation, and the Hargreaves plantar test was used to evaluate thermal sensation. At 12 weeks after transplantation, the von Frey monofilament test scores in the Auto group and in the sensory TP group were significantly higher than those in the NC group (Auto = 2.33 ± 0.21 g, Motor TP = 1.93 ± 0.34 g, Sensory TP = 2.15 ± 0.19 g, NC; 0.94 ± 0.19 g.) (Fig. 3d). For the Hargreaves plantar test for thermal sensation, the Auto group was the only group that had significantly higher scores than those of the NC group (Auto = 19.2 ± 0.5 s, Motor TP = 16.7 ± 1.1 s, Sensory TP = 17.6 ± 0.6 s, NC = 14.4 ± 0.8 s.) (Fig. 3e). These results indicated that transplantation of neurite bundle-derived artificial nerves prevented a reduction in sensitivity and improved some sensory functions.

### Innervated muscle recovery along with nerve regeneration

To evaluate the nerve innervation status of muscles, electromyography (EMG) evaluation was carried out. Sciatic nerve recovery was assessed through electrical nerve stimulation at the proximal side of the injury site with EMG with adequately anesthetized rats. Electrical stimulation was detected in the gastrocnemius muscle on the affected side. (Fig. 3f). Both the motor and sensory TP groups exhibited distinct nerve activity waveforms similar to those of the Auto group, compared to the NC group, which exhibited a minimally detectable waveform. Various degrees of nerve regeneration were detected among all groups, and then the two parameters, latency and amplitude, were quantitatively analyzed. The motor and sensory TP groups showed recovery trends that approached those of the Auto group according to the nerve activity waveform analysis with EMG. However, more numbers would be needed to detect a statistically significant difference. (Latency: Auto = 2.16 ± 0.24 ms, motor TP = 3.01 ± 0.81 ms, sensory TP = 2.77 ± 0.23 ms, NC = 4.25 ± 0.58 ms. Amplitude: Auto = 21.5 ± 2.4 mV, Motor TP = 12.2 ± 1.6 mV, Sensory TP = 14.3 ± 2.0 mV, NC = 6.1 ± 1.0 mV.) (Fig. 3g, h). The extent of muscular atrophy was evaluated by the wet weight of the gastrocnemius muscles at the time of sacrifice at 12 weeks (Fig. 3i). Compared to the NC group, both the motor and sensory TP groups exhibited reduced levels of muscular atrophy (Auto = 0.59 ± 0.03, Motor TP = 0.37 ± 0.02, Sensory TP = 0.45 ± 0.05, NC = 0.28 ± 0.02.) (Fig. 3j). Notably, higher values were observed in the Auto group and the sensory TP group than in the NC group. These findings indicated that the transplantation of artificial nerves, particularly those derived from sensory neurite bundles, facilitated muscle recovery.

### Neurite bundles dose dependency for therapeutic effect

Improvements in motor and sensory function were observed with six neurite bundles. To confirm whether there is a dose-dependent therapeutic effect, we performed transplantation with twice the number of neurite bundles that had previously shown a sufficient functional recovery. No significant differences were observed between 6 and 12 bundles in the sciatic functional index, the von Frey test, the Hargreaves plantar test, EMG analysis (latency, amplitude) and wet weight of the gastrocnemius muscles (Supplementary Figure 3). These results indicate that the therapeutic effect of the artificial nerve is sufficient with six neurite bundles containing several thousand neurites, so that the dose-dependency was not detected.

### Promoting recovery from early transplantation

**Fig. 4:**
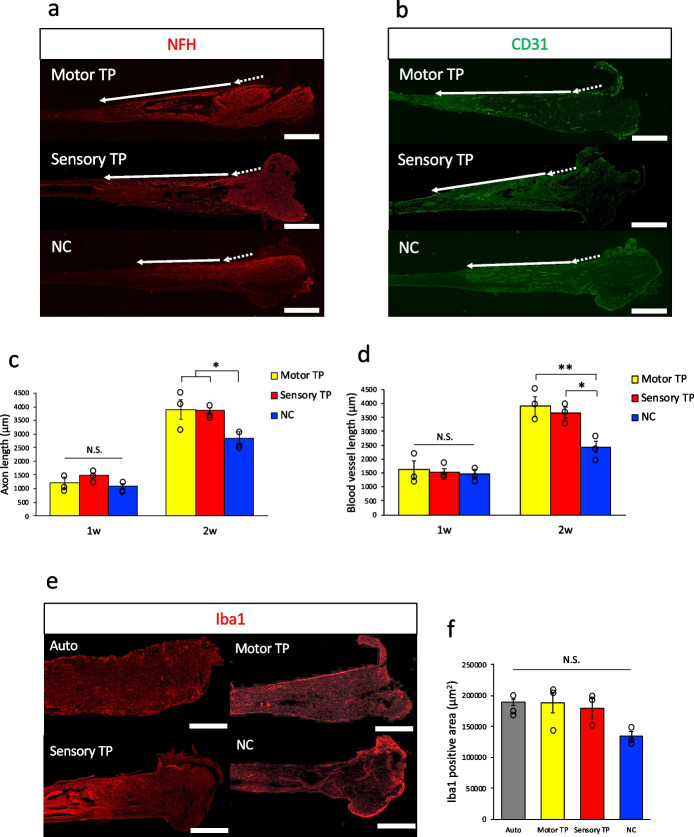
Early-stage tissue evaluation after neurite bundle-derived artificial nerve transplantation. **a**, **b** Immunohistochemistry images with anti-NFH and anti-CD31 antibodies. Dotted arrows indicate the proximal side of the intact nerve areas, and solid arrows indicate the NFH- and CD31-positive regeneration tip in the reconstructed tissue. Scale bars = 1 mm. **c**, **d** Quantitative comparison of axonal extension distance was indicated by NFH-positive areas and that of vascular elongation distance was demonstrated by CD31-positive areas at 1 and 2 weeks after transplantation. Significant differences between groups elongation distances were observed in 2-week samples only. **e**, **f** Iba1 immunohistochemistry image of the proximal part of the regenerated tissue at 2 weeks after transplantation (e). Scale bars = 1 mm. Quantitative analysis of Iba1-positive areas demonstrated that a larger number of macrophages aggregated in the Auto, motor TP and sensory TP (f). (*n* = 3). * *p* < 0.05, ** *p* < 0.01, N.S. = not significant. Data are represented as the mean ± SEM

To analyze the mechanisms by which artificial nerve transplantation promote recovery, early-stage tissues were collected and analyzed after transplantation. Regenerated nerves were harvested at 1 and 2 weeks after transplantation, and the frozen sections were immunostained. Anti-neurofilament-H (NFH) and anti-CD31 antibodies were used to evaluate regenerating axons and recovered blood vessels, respectively (Fig. 4a, b). In the Auto group, the remaining axons and blood vessels were stained throughout the whole autograft tissue, making it difficult to detect the newly regenerated axons and blood vessels (data not shown). To evaluate the regeneration speed by quantifying the regenerated tip portion of the newly formed axons and blood vessels, a comparison was made among the three groups (NC, motor, and sensory TP groups). One week after transplantation, no obvious difference in the length of regenerated axons and vessels was observed between groups (NFH: motor TP = 1211 ± 217 μm, sensory TP = 1471 ± 87 μm, NC = 1083 ± 110 μm. CD31: motor TP = 1609 ± 309 μm, sensory TP = 1544 ± 113 μm, NC = 1471 ± 139 μm.). At two weeks after implantation, the length of regenerated axons (Fig. 4a, c) and regenerated blood vessels (Fig. 4b, d) from the proximal nerve sump edge was increased in the motor and sensory TP group than in the NC group (NFH: motor TP = 3904 ± 359 μm, sensory TP = 3853 ± 106 μm, NC = 2844 ± 224 μm. CD31: motor TP = 3903 ± 349 μm, sensory TP = 3675 ± 192 μm, NC = 2439 ± 210 μm.). These results indicated that the early stage of axonal and vascular outgrowth was accelerated in the motor and sensory TP group starting approximately 1–2 weeks after transplantation. In addition, the Iba1-positive area in the proximal part of the regenerating tissue at 2 weeks tended to be larger in the Auto, motor and sensory TP group than in the NC group (Auto = 189,298 ± 5401 μm^2^, motor TP = 188,050 ± 16,342 μm^2^, sensory TP = 178,855 ± 16,115 μm^2^, NC = 134,438 ± 7796 μm^2^.) (Fig. 4e, f). This evidence indicated that the concentrated macrophages in the proximal portion of the regenerating tissue played important roles in the enhanced recovery group.

### Upregulated expression of macrophage-related immune system genes by expression profiling

**Fig. 5:**
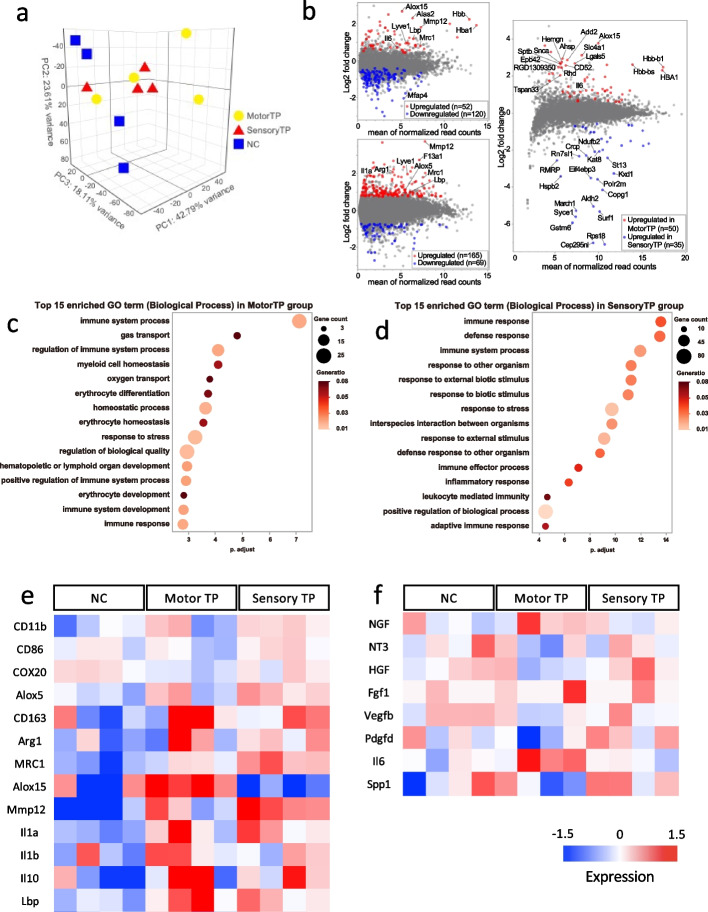
Transcriptome analysis 1 week after neurite bundle-derived artificial nerve transplantation. **a** Principal component analysis of the gene expression data in peripheral nerves at 1 week after transplantation. The spatial arrangement of the points reflects the overall data similarity between the samples. **b** Gene expression MA plots between the following paired group comparison. Red and blue plots indicate differentially expressed genes (*p* < 0.05, fold change > 1.5). Top left: Motor TP vs. NC. Bottom left: Sensory TP vs. NC. Right: Motor TP vs. Sensory TP. **c**, **d**  Dot plot of the top 15 GO biological process terms associated with genes with upregulated expression in the motor TP (c) and sensory TP groups (d) compared with the NC group. **e**, **f**  Heatmap of the expression values (normalized as TPM) of various genes associated with macrophages and regeneration in the motor TP, sensory TP and NC groups

To investigate the molecular mechanism of promoted recovery, transcriptome analysis with next-generation sequencing NGS was carried out on the cells in the nerve conduit one week after artificial nerve transplantation. The principal components analysis (PCA) demonstrated clustering within each group (Fig. 5a). Using MA plots to compare the gene expression levels, both the motor TP and sensory TP groups showed increased expression levels of Mmp12 and Mrc1 compared to those in the NC group (Fig. 5b). Furthermore, we found that M2 macrophage markers such as Alox15 and Arg-1 were upregulated in the motor TP group and the sensory TP group. For genes with more than a 1.5-fold significant difference in expression in the artificial nerve group, Gene Ontology (GO) analysis was conducted (Fig. 5c, d). The analysis identified immunoreaction-related terms such as “immune system development”, “immune system process”, “immune response”, and “adaptive immune response” among the top 15 GO biological process terms. In particular, a larger number of immune-related genes were upregulated in the sensory TP group than in the motor TP group (Fig. 5d). We also created a heatmap to display the genes with significant differences in expression (Fig. 5e, f). In the novel artificial nerve transplantation groups, we observed an upregulation of macrophage-related genes, particularly CD163, Arg-1, Mrc1 and Alox15, which are M2 macrophage markers (Fig. 5e). These results suggested that macrophage activation occurred in the TP groups. The motor TP group showed a selective upregulation of nerve regeneration-related genes such as NGF and Il6, whereas the sensory TP group exhibited more extensive upregulation of genes (Fig. 5f).

In summary, the mechanism of promoted recovery by neurite bundle-derived artificial nerve transplantation is thought to be largely due to the activation of specific macrophages and the upregulation of various nerve regeneration-related genes.

### Insufficient functional and histological recovery in immunodeficient nude rats

From the above-mentioned experiments, the role of immune function and macrophages was found to be quite important for nerve recovery after artificial nerve transplantation with neurite bundles. Therefore, we examined the efficiency of transplantation therapy into the immunodeficient nude rats for evaluating functional changes. The metrics, including SFI, latency and amplitude and gastrocnemius wet weight of Auto, motor TP and NC groups were recorded at 12 weeks after transplantation. No significant differences between the motor TP and NC groups were detected (SFI: Auto =  − 67.7 ± 4.4, motor TP =  − 75.9 ± 1.9, NC =  − 79.6 ± 2.5. Latency: Auto = 1.63 ± 0.13 ms, motor TP = 2.62 ± 0.58 ms, NC = 3.34 ± 0.13 ms. Amplitude: Auto = 24.2 ± 2.12 mV, motor TP = 13.0 ± 1.71 mV, NC = 10.1 ± 4.02 mV. Gastrocnemius muscle wet weight ratio: Auto = 0.71 ± 0.02, motor TP = 0.45 ± 0.02, NC = 0.47 ± 0.04.) (Supplementary Figure 4a-d).

New artificial nerve transplantation into wild-type animals resulted in a behaviorally significant recovery effect in the TP group compared to the NC group (Fig. 3), but no recovery was observed in the immunodeficient animals (Supplementary Figure 4a-d). To confirm the histological difference between the TP and the NC groups, additional detailed evaluation of the regenerated axons was performed between these two groups. Histologically, quantitative analysis of regenerating axons by toluidine blue-stained images of axial sections showed that no significant differences were found between both groups. (Number of axons: TP = 6596 ± 443, NC = 5923 ± 1080. Total area of axons: TP = 29,666 ± 7878 μm^2^, NC = 20,345 ± 4916 μm^2^. Average area of axons: TP = 4.32 ± 0.86 μm^2^, NC = 3.33 ± 0.23 μm^2^.) (Supplementary Figure 4e-h). In the analysis of electron micrographs for nerve regeneration, the approximate straight line of G-ratio values was comparable (TP; y = 0.0265x + 0.6526, NC; y = 0.03x + 0.637.) (Supplementary Figure 4i, j).

These results clearly indicated that artificial nerve transplantation with human neurite bundles to wild-type rats prompted both functional and histological improvements, whereas such improvements were completely diminished in immunodeficient rats. This evidence data emphasizes the important role of immune cells.

### Increased immune cell infiltration associated with artificial nerve transplantation

**Fig. 6:**
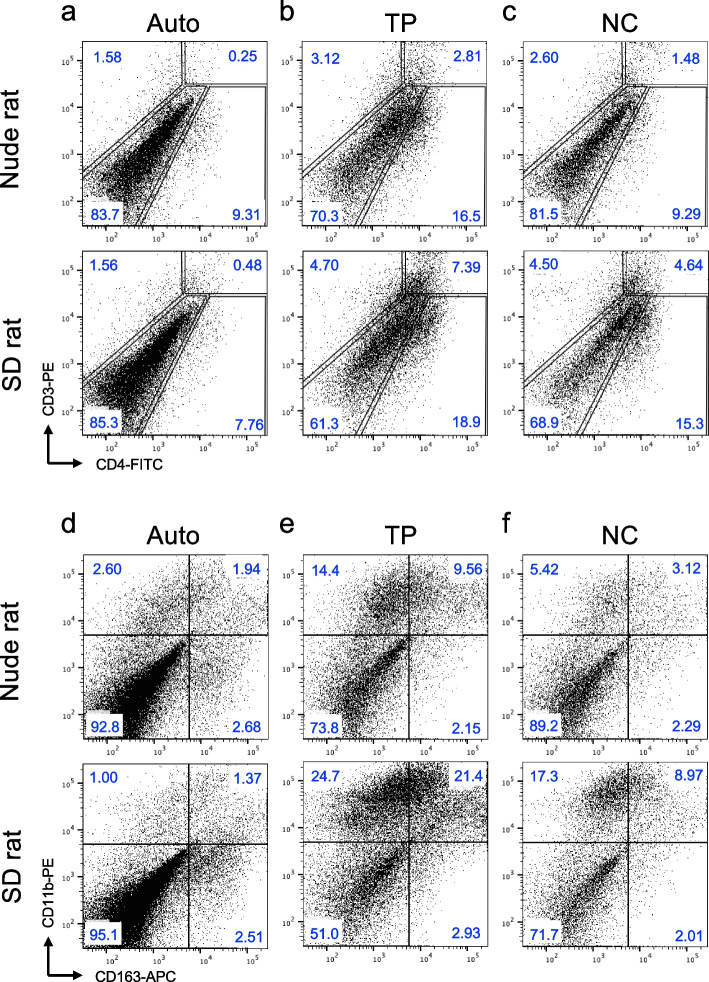
Detection of migrating immune cells in transplanted area. Transplanted areas of the auto, sensory TP, and NC groups at day 7 in nude (top) and SD (bottom) rats were analyzed by flow cytometry. **a**-**c** display FACS profiles of T cell populations present at the transplant site. An electronic gate was placed on hematopoietic cells based on their FSC and SSC scatters, and then the proportions of CD3 + CD4 + (CD4 + T cells) were analyzed. **d**-**f** FACS profiles show the analysis of macrophages present in the transplant area. CD11b + CD163 + cells were defined as infiltrating M2 macrophages

Since superior functional and histologic improvements were observed in wild-type rats after artificial nerve transplantation compared to nude rats, we next evaluated the immunologic status of the transplanted area. Flow cytometric analysis revealed that the proportions of CD4^+^ T cells in the tissues from the sensory TP group were increased compared with the other groups (auto and NC) (Fig. 6a-c). The proportion of CD4^+^ T cells in the tissues from the TP group in wild-type rats was higher than that from the TP group in nude rats (Fig. 6a-c). At the same time, the proportions of CD11b^+^ macrophages in the tissues from the TP group and the NC group, especially in the TP group, were increased in wild-type rats (Fig. 6d-f). Interestingly, the percentage of CD163^+^ M2 macrophages among CD11b^+^ macrophages was increased in tissues from the TP group in wild-type rats (Fig. 6d-f).

### Assessment of cell survival and tumor growth potential after transplantation

**Fig. 7:**
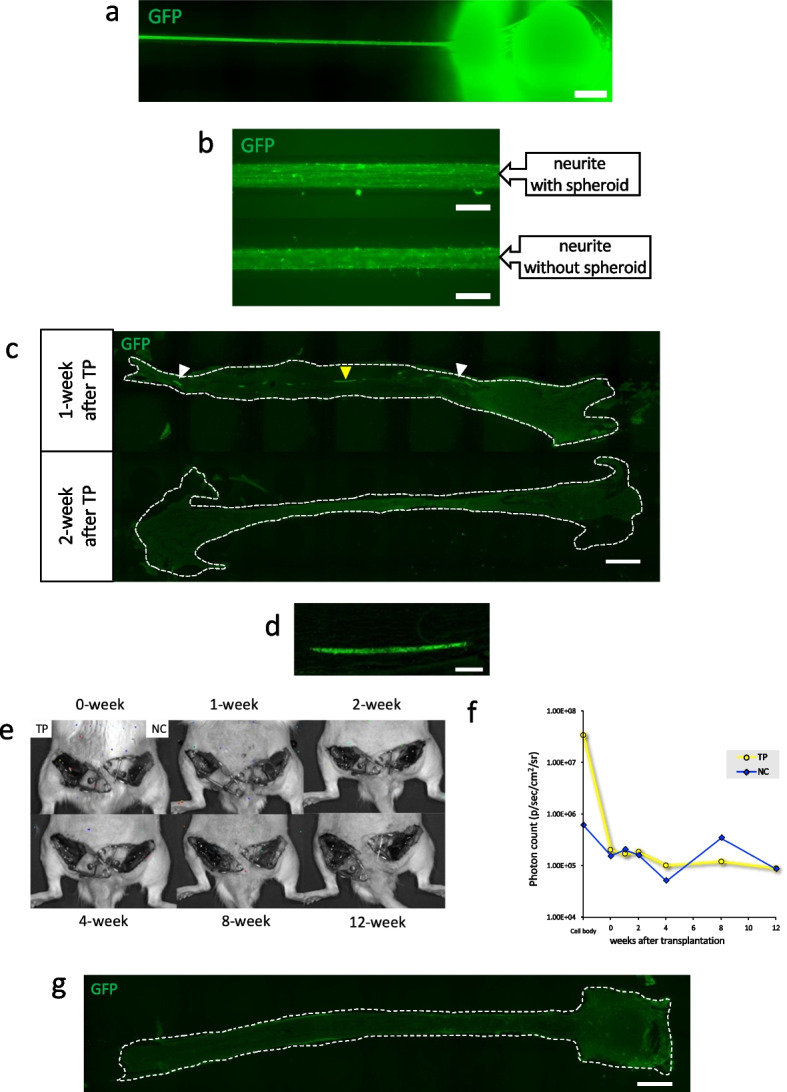
Evaluation of cell survival and tumor growth derived from neurite bundle transplantation. **a** Immunofluorescence images of membrane-bound GFP-labeled nerve organoids in the microfluidic device clearly visualized the green neurite bundles with anti-GFP antibody immunostaining. Scale bar = 300 μm. **b** Immunofluorescence images of neurite bundles labeled with membrane-bound GFP confirmed by anti-GFP antibody staining. Upper: Neurite bundles cultured with spheroids. Bottom: Neurite bundles cultured without spheroids for 2 weeks. Scale bars = 100 μm. **c** Immunofluorescence images of regenerated tissue of membrane-bound GFP-labeled nerve bundles at 1 and 2 weeks after implantation, stained with anti-GFP antibody. White and yellow arrowheads: neurite bundle human tissue was positively stained; Scale bar = 1 mm. **d** Magnified image of the regenerating nerve tissue with transplantation (yellow arrowhead). Scale bar = 100 μm. **e** Live cell tracking after implantation of ffLuc-labeled artificial nerves. No bioluminescence reactivity was observed at any time point from 0–12 after transplantation. Left: transplanted side. Right: healthy control side. **f** Quantitative analysis of the photon counts derived from the ffLuc-labeled transplanted tissue up to 12 weeks. **g** Immunofluorescence image of a sagittal section of regenerated tissue at 12 weeks after transplantation, and no fluorescence-positive areas were observed. Scale bar = 1 mm

To address the biosafety of artificial nerves with neurite bundles, in vivo cell survival and tumor formation were analyzed over time. First, neurite bundles were labeled using membrane-bound GFP (mGFP) by lentivirus infection (Fig. 7a). The mGFP fluorescence of the neurite bundles at two weeks after spheroid removal was almost the same as that of the neurite bundles maintained with spheroids (Fig. 7b). This finding showed that the neurite bundles were still intact 2 weeks after spheroid removal. Neurite bundles labeled with mGFP were transplanted into the injured sciatic nerve, and the nerve was harvested at 1 and 2 weeks after transplantation for histological investigation. By staining with anti-GFP, linear fluorescence-positive lines could be observed in the transplanted nerve conduits at 1 week posttransplantation, but the fluorescence level was drastically reduced at 2 weeks (Fig. 7c, d). These results indicated that most of the transplanted hiPSC-nerve organoid-derived neurite bundles disappeared approximately 2 weeks after transplantation in wild-type rats. The advantage of the novel artificial nerves is that they are created by excision of the spheroids, which reduces the risk of tumor formation after transplantation. We ensured that no dividing cells in nerve organoids were contaminated during the creation of the artificial nerve by labelling them with ffLuc (genetically modified green fluorescence protein (cpVenus) fused with firefly luciferase gene) [20], and cell survival and proliferation were tracked with the In Vivo Imaging System (IVIS). If the cells were viable and proliferated, bioluminescence could be detected by IVIS (Supplementary Figure 5a). The cells were labeled by ffLuc lentivirus during culture and before creating the novel artificial nerves, and it was confirmed by IVIS that only the cell body emitted light and that no light was emitted in the neurite portion (Supplementary Figure 5b). No obvious cell body contamination was observed in the neurite bundles during culture. The artificial nerves were transplanted into the injured sciatic nerve, and the implanted area was opened to check bioluminescence every 1 to 4 weeks until 12 weeks. From 0 to 12 weeks after implantation, no obvious bioluminescence signals were detected (Fig. 7e). Luminescence signals were quantitatively analyzed, and the signals of the neurite bundle-derived artificial nerve grafts were comparable to those of the healthy side (Fig. 7f). At 12 weeks after transplantation, the nerves were harvested and stained with anti-GFP, but no stained areas were observed (Fig. 7g). In addition to no fluorescence-positive areas in the ffLuc-labeled neurite bundle-transplanted animals, no tumorigenesis was detected. This solid evidence clearly indicated that there was no obvious cell body contamination of the neurite bundles in artificial nerves and completely no neoplastic growth over time after transplantation.

## Discussion

Here, we report that hiPSC-derived neurite bundle artificial nerve implantation improves the motor and sensory function of rat sciatic nerves with extensive defects. Transplantation of motor and sensory neurite bundles resulted in great histological recovery, including as axonal regeneration, myelination, and angiogenesis, and the results confirmed the biosafety of the artificial nerves. The transplantation was especially effective in improving sensory function, and the autotomy score exceeded that of autograft nerve transplantation. Previous reports have shown that the number of injuries decreased in groups treated with various artificial nerves [21, 22]. Remarkably, none of the transplanted animals in this experiment showed any loss of fingers or even nails. Since the nerve grafts usually used for autologous nerve transplantation are sensory nerves, these new artificial nerves that imitate them are expected to be incredibly useful.

Commercially available artificial nerve conduits are filled with collagen, which means the artificial nerves used to clinically treat patients are very similar to those in the NC group in this study. Since novel artificial nerves showed drastically higher efficacy than the NC group treatment in all evaluation systems, it is likely that these novel artificial nerves will exhibit increased efficacy in nerve regeneration compared to that of the existing artificial nerve. In summary, our novel artificial nerve is a promising transplantation therapy candidate for peripheral nerve injury.

In our analysis, the sensory TP group showed a significantly higher therapeutic effect than the motor TP group in some evaluations, and we considered the causes. Comprehensive immunostaining results of motor and sensory markers demonstrated the heterogeneity of the differentiated neurons from iPSCs in the spheroids. Therefore, the neurite bundles also had heterogeneous properties. It is very difficult to make a direct comparison because it is slightly different from the transplantation of nerves in human clinical practice, and one study has reported that nerve recovery is improved by using motor nerves as autograft nerve grafts [17]. One reason for this notion may be that the diameter of the Schwann cell basal lamina tube is larger in motor nerves than in sensory nerves, allowing more regenerating nerve fibers to pass through the graft. Although it was contrary to previous reports, our experimental results indicated the superiority of the sensory TP group and demonstrated the scientific basis for performing sensory nerve transplantation in human clinical practice. Since the artificial nerve in this study consists of unmyelinated neurite bundles and does not contain Schwann cells, it is unlikely that its functional improvement was due to the Schwann cell basal lamina canal. Even with unmyelinated nerves, the sensory TP group, consisting of large-diameter neurites with many neurofilaments, promoted improvement in nerve function in transplantation experiments. Larger diameter groups, whether myelinated or unmyelinated, may be more helpful in inducing appropriate immune responses to promote nerve regeneration.

In the novel artificial nerve transplantation group, enhanced functional and histologically recovery was observed in wild-type rats but not in immunodeficient rats. A previous report showed that the axonal regeneration induced by acellular allografts was attenuated in immunodeficient, T-cell-deficient rats [23]. Our results were consistent with this report. In addition, an enlarged Iba1-positive area was observed at the proximal part of the regenerated tissue after the novel artificial nerve transplantation in wild-type rats. Based on the results of these experiments, we considered that the therapeutic effect of novel artificial nerve transplantation was mediated by the immune system, particularly by T cells and macrophages. A previous study showed that once macrophages sense the hypoxic status of the nerve defect area, macrophages induce the rearrangement of vascular endothelial cells and promote angiogenesis [24]. Thereafter, Schwann cells migrate along these blood vessels and form Büngner’s bands, which act as a scaffold for axon regeneration. Additionally, it was also reported that macrophages play important roles in promoting remyelination by regulating the maturation of Schwann cells after nerve injury [25]. Based on previous reports and the results of our experiments, it was implied that the transplanted neurite bundles induced the recruitment of macrophages and supported enhanced host angiogenesis, which subsequently promoted host axonal regeneration and myelination by controlling the condition of Schwann cells.

The results from the transcriptome analysis showed upregulated expression of macrophage-related genes in the artificial nerve transplant group, and the results from GO analysis also showed upregulation of immune-related genes. In particular, the expression of M2 macrophage markers such as Alox15, Mrc1 and Arg-1 was significantly upregulated [26–28]. These results support the hypothesis that artificial nerves contribute to functional improvement via macrophages. As shown in the GO analysis, a larger number of immune-related genes were upregulated in the sensory TP group than in the motor TP group. In addition, many neuroprotective genes were upregulated in the sensory TP group, but a limited number of nerve regeneration-related genes, such as Il6, were only slightly upregulated in the motor TP group [29]. Extensive activation of the immune system and of neuroprotective genes might be the key mechanisms responsible for the differences in treatment efficacy between groups.

The typical polarity of macrophages is often defined as M1 and M2 macrophages to understand their diversity and plasticity [30]. The cytokines such as IL-4 and IL-13 produced by type 2 T helper (Th2) cells, a subset of Th cells in CD4^+^ T cells, are known to induce M2 macrophage polarization [31]. M2 macrophages play a key role in wound healing through their productions of growth factors such as TGF-β and platelet-derived growth factor [32]. Previous reports have also demonstrated the involvement of immune responses mediated by CD4^+^ T cells, including T helper (Th) cells, regulatory T cells (Tregs), and M2 macrophages, in the process of nerve regeneration [33]. In this study, we demonstrated the improvement of nerve regeneration accompanied by the higher CD4^+^ T cell and M2 macrophage infiltration by novel artificial nerve transplantation compared with NC group observed in wild-type rats. These results suggest that the induction of CD4^+ ^T cells and M2 macrophages are involved in the effective nerve regeneration by novel artificial nerve transplantation. Th2 cell polarization during nerve regeneration improves functional recovery and myelination [34]. In addition, IL-4 and IL-13 produced by Th2 cells facilitate M2 polarization of macrophages to promote nerve regeneration [35]. Although the detailed subset of Th cells among the CD4^+^ T cells induced by the novel artificial nerve transplantation observed in this study is unclear, it is possible that they include Th2 cells to effectively promote nerve regeneration. In contrast, we showed that the infiltrations of CD4^+^ T cells and M2 macrophages, and the functional and histological nerve regeneration were suppressed by the artificial nerve transplantation in nude rats, which are severely deficient in T cell function, compared with wild-type rats. Such a reduced accumulation of M2 macrophages at the site of artificial nerve transplanted in nude rats compared to that in wild-type rats may be due to the inhibition of M2 macrophage polarization by their limiting T cell function. Meanwhile, autologous nerve transplantation, in which accelerated nerve regeneration was observed, did not show the marked infiltration of CD4^+^ T cells and M2 macrophages. These results may be due to the possibility that the artificial nerves have higher immunogenicity than autologous nerves as well as the differences in immune response-dependent mechanisms between nerve regeneration by artificial nerve transplantation and that by autologous nerve transplantation. In addition, nerve regeneration associated with such immune cell inductions was promoted in the artificial nerve transplantation group compared with the NC group, suggesting the involvement of such immune responses in the improvement of nerve regeneration by artificial nerve transplantation. Taken together, our results suggest that CD4^+^ T cell, which may include Th2 cells, -dependent induction of M2 macrophages is crucial for nerve regeneration induced by artificial nerve transplantation.

Autologous nerve transplantation is the first-line treatment for peripheral nerve injury; however, allogeneic nerve transplantation is allowed in several countries and has achieved certain therapeutic results. The xenograft transplantation results in this study showed that human-derived neurite bundle transplantation into wild-type rats was effective, indicating the possible application of the novel artificial nerve without triggering immunosuppression. It was suggested that it was possible to transplant the allograft nerve derived from hiPSCs into a host patient while retaining normal immune function. The great advantage of allogeneic nerves is that it is possible to prepare them in advance using neurite outgrowths generated in advance by hiPSCs, which can proliferate indefinitely. In contrast, generating autografts using the patient's own iPSCs is a slow process. If allogeneic nerve transplantation using neurite bundles derived from iPSCs originating from other cells is allowed, a ready-to-use stock could be prepared to quickly treat nerve injury.

Next, we will address the safety of these novel artificial nerves. Neural crest lineage cells, including Schwann cells, can be induced from hiPSCs, and transplantation of neural crest cells into injured peripheral nerves has been reported to promote nerve regeneration [36–38]. However, the risk of tumorigenesis is always an issue when using hiPSCs for cell transplantation therapy [39, 40]. The risk of tumor formation in the novel artificial nerve was greatly reduced by the removal of all cell bodies in the spheroid. No tumorigenesis was detected in any of the transplanted animals (more than 50 samples), with a long follow-up time, and no cell proliferation could be detected by ffLuc lentivirus luminescence labeling. By using membrane-bound GFP, we found that transplanted human neurites completely disappeared in approximately 2 weeks. Confirming the safety of this novel artificial nerve demonstrated the possibility of its clinical application. As mentioned above, acellular allograft transplantation is optimal because the host immune function is maintained. In other words, there is no need to use immunosuppressants at the time of transplantation, so clinical complications can be avoided, just as with bedside human allograft nerve transplantation. Another major advantage of acellular allogeneic transplantation is that the contaminated cells would be removed, even if the cells are derived from hiPSCs, ensuring a high level of safety. Tumor formation is often a concern when using hiPSCs, but this risk is greatly reduced with the use of these new artificial nerves.

Finally, although our artificial nerves have been shown to be therapeutically effective and safe, there are still some issues to be addressed. In this study, a silicone tube was used for the outer cylinder; however, it is not practical for clinical application. In near future, the best fit outer conduit will need to be created made of bioabsorbable materials. Additionally, when considering clinical applications, it is necessary to invent longer artificial nerves for larger defects because of the size limitation of the rodents used in this experiment. Therefore, it will be necessary to conduct experiments using larger animals to verify the therapeutic effects and confirm biosafety.

## Materials and methods

### Motor and sensory spheroid preparation derived from human iPSCs

Frozen motor neurons generated from human iPSC-derived cells (iXCells Biotechnologies, Inc.) were thawed, and then dead cells were removed with reagents (EasySep Dead Cell Removal (Annexin V) kit, STEMCELL Technologies) after centrifugation to enhance the purity of live cells. More than 75% live cells were placed in a 96-well V-bottom plate at a density of 4 × 10^4^ cells/well, cultured at 37 °C in 5% CO_2_ for 1 week, cultured in motor neuron maintenance medium (iXCells Biotechnologies, Inc.) that was changed every 3 days, and formed into motor cell spheroids in 1 week. Frozen sensory neurons differentiated from human iPSC-derived sensory neurons (AXOL Bioscience Ltd.) were thawed, and then dead cells were removed with reagents (EasySep Dead Cell Removal (Annexin V) kit, STEMCELL Technologies) after centrifugation to enhance the purity of live cells. More than 80% live cells were placed in a 96-well V-bottom plate at a density of 4 × 10^4^ cells/well and were cultured in Neural Plating Medium (AXOL Bioscience Ltd.) and 10 μM Y27632 (Nacalai tesque) for 24 h. The cells were treated with sphere-forming medium: Neurobasal Plus medium (Thermo Fisher Scientific) supplemented with B-27 Plus Supplement (Thermo Fisher Scientific), penicillin‒streptomycin (Thermo Fisher Scientific), 20 ng/mL BDNF (GenScript), 20 ng/mL GDNF (GenScript), 10 ng/mL βNGF (PeproTech), and 20 ng/mL NT-3 (PeproTech) for 2 days. The cells were treated with medium containing 2.5 µg/mL mitomycin C for 2 h, and the medium was replaced with sphere-forming medium for 3 more days. The medium was changed every day, and the cells were formed into neuronal spheroids at 37 C in 5% CO_2_ [11].

### Generation of neurite bundle-derived artificial nerve

The basic procedure for generating nerve organoids was previously described [11, 12]. After coating the bottom surface of the organoid culture chip (a microfluidic device that has two culture compartments and one channel in between), a spheroid was placed in the spheroid compartment. Approximately 2 cm neurites were formed from the spheroids by culturing the spheroids in Neurobasal Plus medium (Thermo Fisher Scientific) supplemented with B-27 Plus Supplement (Thermo Fisher Scientific), penicillin‒streptomycin (Thermo Fisher Scientific), 20 ng/mL BDNF (GenScript), and 20 ng/mL GDNF (GenScript) at 37°C in 5% CO_2_ for approximately 3 to 4 weeks. The medium was changed twice a week. The neurites were removed from the organoid culture chip and linearly arranged in a collagen solution (beMatrix collagen AT, Nitta gelatin, Osaka, Japan) on a cool plate. The collagen solution with several neurite bundles was gelled by incubation at 37 °C for 40 min. Immediately after resecting the neural spheroid in which the cell bodies were located, the neurite bundle-containing collagen gel was placed in a 15 mm-long silicone tube (inner diameter: 1.5 mm, outer diameter: 2.0 mm, Taiyo Kogyo, Tokyo, Japan).

### Experimental animals and surgical procedures

SD rats (8 weeks old, males, Japan SLC Inc., Shizuoka, Japan) and immunodeficient F344/NJcl-*rnu/rnu* nude rats (8 weeks old, males, CLEA Japan, Tokyo, Japan) were used to prepare a previously described sciatic nerve injury model [38]. This animal experiment was approved by the Keio University and Niigata University Institutional Animal Care and Use Committee in accordance with the Institutional Guidelines (approval number: 17024-(2) in Keio and SA01058 in Niigata). All rats were deeply anesthetized with a subcutaneous injection of a mixture of three anesthetics (2.5 mg/kg butorphanol, 0.4 mg/kg medetomidine hydrochloride, and 2 mg/kg midazolam).

A dorsal longitudinal skin incision was made, and the sciatic nerve was exposed by incising the gluteal fascia and bluntly splitting the gluteal muscle. The sciatic nerve was resected at the middle of the thigh. The gap was repaired by fixing the nerve stumps 1 mm inside the end of the artificial nerve tube using a horizontal mattress suture of 9–0 monofilament nylon at each end, leaving a 13 mm interstump gap. The rats were assigned to four groups as described below. Rats in the transplantation group received a silicone tube filled with artificial motor or sensory neurite-containing collagen gel to bridge the gap (Motor or Sensory TP 6 bundles group: 6 SD rats each, Motor TP 12 bundles group: 4 SD rats and 4 nude rats). Rats in the negative control group received a silicone tube filled with only collagen gel to bridge the gap (NC group: 7 SD rats and 4 nude rats). In the autograft group, the 13 mm gap was reconstructed by turning the resected nerve over and bridging the resected nerve (Auto group: 6 SD rats and 4 nude rats). All procedures of suturing nerves were carried out under a surgical microscope. The overlying muscle layers and skin were sutured with 5–0 nylon sutures to close the surgical site. Twelve weeks after transplantation, nerve regeneration was assessed by electrophysiology, and muscle recovery was assessed by wet gastrocnemius muscle weight. All rats in each group were euthanized, and the conduits were harvested.

### Luminescence measurement

Using a live cell tracking analysis to check for neoplastic growth, cell survival in artificial neurite bundles on organoid culture chips and after transplantation in rats was evaluated. A luciferase substrate, D-luciferin (Summit Pharmaceuticals International Corporation, Tokyo, Japan), was intraperitoneally injected into the rats (0.3 mg/g body weight), and the fluorescence (cpVenus) and luminescence (firefly luciferase) fusion protein ffluc-labeled artificial neurite was monitored using an In Vivo Imaging System (IVIS)-spectrum and CCD optical macroscopic imaging system (Caliper Life Sciences, MA, USA) [20]. Bioluminescent signals of cell bodies and neurites of the neural organoids in the chamber before creating artificial nerves were each measured. Bioluminescence signals of neurite bundles after transplantation were measured until 12 weeks after transplantation.

### Lentiviral vector construction of mGFP, the membrane-bound form of EGFP

The mGFP sequences from pCAG-mGFP (Addgene) were cloned and newly inserted under the EF1 promoter sequence [41]. Recombinant lentiviral vectors were produced by transient transfection of three plasmids, pCAG-HIVgp, pCMV-VSV-G-RSV-Rev, and the lentiviral vector plasmid (CSII-EF-mGFP), into HEK293T cells as previously described [42, 43].

Culture supernatant containing lentivirus was concentrated by ultracentrifugation (50,000 G for 2 h at 4 ºC), and the viral pellets were resuspended in HBSS (14,025,092; Thermo Fisher Scientific). Harvested lentiviruses were stored at -80 ºC before use. The multiplicity of infection (MOI) values were adjusted to 15 based on the total cell numbers.

### Motor functional evaluation for gait analysis, ankle motion range and muscle weight

The motor function of the rats’ lower extremities was evaluated every two weeks using the DigiGait System (Rat Specifics, Inc., Framingham, MA, USA). Rats were placed on a transparent board, and their footprints were scanned every two weeks after transplantation. We measured the sciatic functional index (SFI), which was calculated using the following formulation [44]: SFI =  − 38.3 × (EPL − NPL/NPL) + 109.5 × (ETS − NTS/NTS) − 13.3 × (EIT − NIT/NIT) − 8.8 (EPL: experimental print length, NPL: normal print length, ETS: experimental toe spread, NTS: normal toe spread, EIT: experimental intermediary toe spread, NPL: normal intermediary toe spread). If the assessor was unable to measure any parameter due to a toe injury, a fixed lowest value of SFI =  − 100 was used [45]. The ankle motion range was evaluated when the rats were adequate anesthesia, and the degree of ankle passive motion range on the operated limb was measured at 12 weeks after surgery. Muscle weight recovery was measured at 12 weeks after transplantation. The bilateral gastrocnemius muscles were harvested, and muscle recovery was calculated by comparing the wet weights between the limbs of the experimental and control rats.

### Autotomy score

At 12 weeks after transplantation, the autotomy score was measured to assess reduced sensitivity. The scores were counted as follows [46]: A score of 1 was given for the removal of 1 or more nails. An additional score of 1 was added for each injured distal half digit. A further score of 1 was added for each injured proximal half digit. The highest score reached 11 if all of the nails and toes were injured.

### Touch sensation and thermal sensation assessment

The von Frey hair test was performed to assess tactile hyperalgesia using a monofilament (Semmes‒Weinstein Aesthesiometer; Stoelting, Wood Dale, IL, United States). Rats were placed in boxes with mesh flooring and were allowed to acclimatize for at least 30 min before sensory behavior assessment. Mechanical hyperalgesia was tested by applying gradually increasing pressure to the mid-plantar surface of both hind paws with nylon monofilaments. The stimulus was applied manually to the mid-plantar surface of the hind paw and continued until withdrawal [47]. The up-down method was used to calculate the threshold [48]. The Hargreaves plantar test was carried out to assess thermal sensation using a specific apparatus (IITC Life Science, Woodland Hills, CA, United States). The time to the withdrawal response after the application of radiant heat to the plantar surface of the hind paw was measured, and the average latency across three trials per paw on each side was recorded [49].

### Electrophysiological study

Electrophysiological evaluation was performed using an electromyography apparatus (MEB-9402; Nihon Kohden, Tokyo, Japan) as previously described [50]. With an adequately anesthetized rat, the sciatic nerve was re-exposed at 12 weeks after transplantation, and electrical stimuli (single-pulse shocks, 1 mA, 0.1 ms) were applied to the intact sciatic nerve proximally 5 mm from the graft. Waveforms were detected in the gastrocnemius muscle. To evaluate the functional recovery of nerves, EMG wave patterns were captured with electromyography. The parameters of waves, such as the amplitude and the latency, were analyzed.

### Fluorescence immunohistochemistry

The cell bodies in the spheroids that were removed from the neurites were immunohistochemically analyzed to evaluate the properties of the transplanted nerve fibers. Sciatic nerves were harvested from deeply anesthetized rats at 1, 2, 8, and 12 weeks after transplantation for histological analysis. Samples were fixed in 4% PFA in 0.1 M PBS at 4°C for 24 h and placed in 15% and 30% sucrose for cryoprotection. Silicone tubes surrounding the regenerating nerves were removed during fixation. The samples were embedded in a frozen section compound (FSC22 Blue; Leica Biosystems, Germany), frozen in liquid nitrogen and axially and sagittally sectioned at a thickness of 10 µm by a cryostat (CM3050S; Leica Microsystems, Wetzlar, Germany). After blocking with the blocking solution, Blocking One (Nacalai Tesque, Kyoto, Japan) 1:20 dilution with 0.1 M PBS and 0.2% Triton-X100, primary antibodies were diluted in the blocking solution. The primary antibodies used in this study were human polyclonal anti-pan-ELAVL (ELAV-like protein 2/3/4) antibody (1:2000, kindly provided from Prof. Robert Darnell, Rockefeller University), rabbit polyclonal anti-TrkA antibody (1:500, kindly provided from Prof. Louis F. Reichardt, UCSF), chicken polyclonal anti-TrkB antibody (1:500, kindly provided from Prof. Louis F. Reichardt, UCSF), goat polyclonal anti-TrkC antibody (AF373, 1:200, R&D systems), rabbit polyclonal anti-parvalbumin antibody (PV-28, 1:1000, Swant), rabbit polyclonal anti-CGRP antibody (BML-CA1134, 1:500, Enzo Life Sciences), Goat polyclonal anti-Choline acetyltransferase antibody (AB144P, 1:200, Chemicon), Mouse monoclonal anti-Islet1 and Islet2 antibody (39.4D5, 1:200, DSHB), rabbit polyclonal anti-Neurofilament heavy polypeptide antibody (ab8135, 1:500, Abcam), goat polyclonal anti-mouse and anti-rat CD31 (AF3628, 1:100, R&D), rabbit polyclonal anti-Iba1 antibody (GtX100042, 1:500, GeneTex), goat anti-GFP antibody (600–101-215, 1:1000, Rockland). The sections were incubated with Alexa Fluor-conjugated secondary antibodies (1:800; Thermo Fisher Scientific). Nuclei were stained with Hoechst 33,258 (94,403, 10 µg/ml; Sigma‒Aldrich). The fluorescence signals were visualized with a fluorescence microscope (BZ-X800; Keyence, Osaka, Japan) or a confocal laser-scanning microscope (LSM780; Carl Zeiss, Jena, Germany). All image analyses were performed using ImageJ software.

### Electron microscopic histological analysis

The motor and sensory neurites bundles before transplantation (*n* = 5 each) and the operated sciatic nerves from the NC (*n* = 7), motor TP (*n* = 5), sensory TP (*n* = 4) and Auto groups (*n* = 5) were prepared for electron microscopic observation, as previously described [51]. Briefly, samples were dissected and fixed with 2.5% glutaraldehyde in 0.1 M sodium cacodylate buffer (pH 7.4) for 24 h at 4°C. After two hours of postfixation with 1% osmium tetroxide at 4 ºC, the samples were dehydrated using a graded series of ethanol concentrations, acetone, and n-butyl glycidyl ether (QY-1, Okenshoji Co. Ltd., Japan), infiltrated with graded epoxy resin mixtures (epoxy resin/QY-1 = 1:3, 1:1, 3–1) and 100% epoxy resin, and finally embedded in 100% epoxy resin followed by polymerization for 72 h at 65 ºC. Semithin sections at 1 μm thickness and ultrathin sections at 70 nm thickness in the axial plane were prepared from resin blocks by an ultramicrotome (UC7, Leica, Germany). Semithin sections were stained with 0.1% toluidine blue and imaged with a microscope (BZ9000; Keyence, Osaka, Japan) for quantification of the total axon number and area using ImageJ software. Ultrathin sections were collected on copper grids and silicon wafers, stained with uranyl acetate and lead citrate, and imaged under transmission electron microscopes (JEM-1400Plus, JEOL Ltd., Tokyo, Japan, and H-7650, Hitachi) and scanning electron microscopes (SU6600 and S-3700 N, Hitachi High Technology, Tokyo, Japan, multiSEM505, CarlZeiss, Oberkochen, Germany). The myelin thickness was evaluated by G-ratio analysis, which is calculated by dividing the diameter of the axon by the outer diameter of myelin. A total of 120 randomly selected myelinated axons from the scanning electron microscopy images per animal were analyzed by using Myeltracer software [52]. For quantification of axonal diameter, the minor axis diameter and density of neurites only with the neurites over 0.8 circularity were measured from 10 independent TEM images with using Image-J and JMP (JMP Statistical Discovery, version 17, SAS Institute Inc.) software.

### Transcriptome analysis with NGS

Regenerated tissue one week after transplantation were used as samples. Total RNA was isolated from each sample using the miRNeasy Mini Kit (QIAGEN, Valencia, CA, USA). The integrity and quantity of the total RNA were measured with an Agilent 2100 Bioanalyzer and RNA 6000 Pico Kit (Agilent Technologies, Inc., Santa Clara, CA, USA). Total RNA obtained from each sample was subjected to sequencing library construction using the TruSeq Stranded mRNA Library Prep Kit (Illumina, Inc., San Diego, CA, USA) according to the manufacturer’s protocols. The equally pooled libraries of the samples were sequenced using NovaSeq 6000 (Illumina, Inc.) in 101-base-pair (bp) paired-end reads. Sequencing adaptors, low-quality reads, and bases were trimmed with the Trimmomatic-0.39 tool [53]. The sequence reads were aligned to the rat reference genome (rn6) using STAR 2.7.9a [54]. The aligned reads were subjected to downstream analyses using StrandNGS 4.0 software (Agilent Technologies, Inc.). The read counts allocated for each gene and transcript (RefSeq Database 2016.05.11) were quantified using a transcripts per million (TPM) method [6, 55]. Statistical analysis between two groups was performed using a moderated t test [56]. The differentially expressed genes (DEGs) were estimated to be significant if the FC (fold change) > 1.5. To summarize the biological aspects of the DEGs, we employed Gene Ontology (GO) analysis [57]. The GO analysis calculates the probability that a gene from the analysis will correspond by chance to a list of genes categorized by GO. The GO analysis was performed using Strand NGS.

### Flow cytometry

The tissue of transplanted area was minced with a scissors, after which the tissue fragments were incubated for 1 h at 37 ºC in Dulbecco's Modified Eagle Medium (DMEM: Invitrogen) in the presence of 0.2% collagenase (Wako Chemicals, Cell dissociation grade), and 25 μg/ml deoxyribonuclease (Sigma-Aldrich). The suspension was filtered through a cell strainer (Falcon 2350, 70 μm) to remove debris and tissue fragments, after which the cells were pelleted by centrifugation at 300 g for 5 min at room temperature. Next, the cells were resuspended in calcium- and magnesium-free Hank's Balanced Salt Solution (HBSS) (Fujifilm) supplemented with 2% FBS, 10 mM HEPES and 1% penicillin/streptomycin (P/S). The cells were stained with immune cell markers as described below; PE conjugated mouse anti-rat CD3 (BD Pharmingen, Clone G4.18, Catalog No:554833), FITC conjugated mouse anti-rat CD4 (BD Pharmingen, Clone OX-35, Catalog No:554837), Catalog No:561588), PE conjugated mouse anti-rat CD11b/c (BD Pharmingen, Clone OX-42, Catalog No:554862), and Alexa Fluor® 647 conjugated mouse anti-rat CD163 (Bio-Rad Laboratories, Inc., Clone ED2, Catalog No: MCA342A647). Flow-cytometric analysis was performed on FACSMelody (BD Biosciences), and the data were analyzed using FlowJo software (BD Biosciences).

### Statistical analysis

Statistical analyses were performed using SPSS statistics (Japan IBM, Tokyo, Japan, v.26.0.0.0). All recorded data are presented as the mean ± SEM. For analysis, the Tukey‒Kramer test was performed among the 4 groups. p values of < 0.05 indicate statistical significance (* *p* < 0.05 and ** *p* < 0.01).

### Supplementary Information

**Additional file 1: Figure S1.** Microfluidic device used to create the nerve organoids with spheroids and neurite bundles.

** Additional file 2: Figure S2.** Images of ankle joint ROMunder anesthesia at 12 weeks.

** Additional file 3: Figure S3.** There was no significant difference between the transplantation group with 6 neurites’ bundles and that of 12 bundles in the recovery of motor and sensory functions.

** Additional file 4: Figure S4.** Limited functional and histological recovery in immunocompromised rats.

** Additional file 5: Figure S5.** Bioluminescence live cell imaging experiments with IVIS.

## Data Availability

The datasets are available from the corresponding author on reasonable request.
