# Supplementary material for: Novel artificial nerve transplantation of human iPSC-derived neurite bundles enhanced nerve regeneration after peripheral nerve injury
Source: Inflamm Regen. 2024 Feb 13;44:6. doi: 10.1186/s41232-024-00319-4 (PMC10863150; doi:10.1186/s41232-024-00319-4)
Supplement: Supplementary file 1 — Additional file 1: Figure S1. Microfluidic device used to create the nerve organoids with spheroids and neurite bundles. [file 41232_2024_319_MOESM1_ESM.pdf]

Supplementary Figure. 1

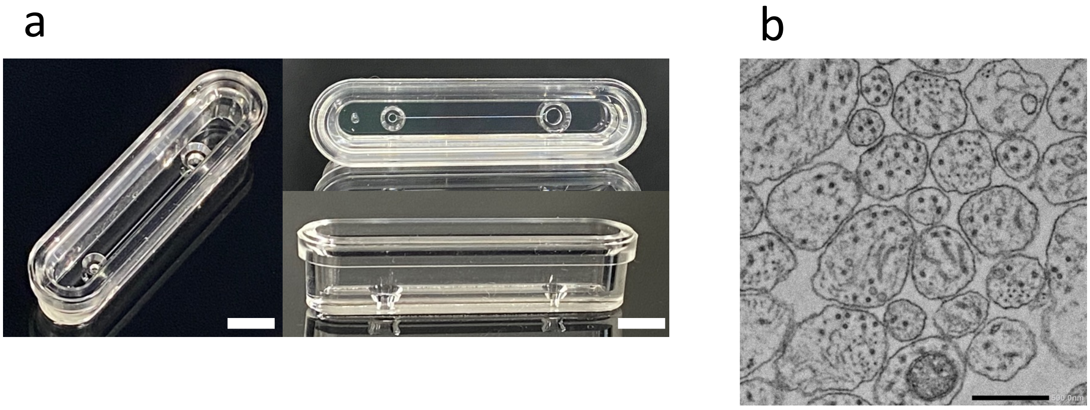

Supplementary Figure. 1

Microfluidic device used to create the nerve organoids with spheroids and neurite bundles.

a, Appearance of the microfluidic device from different aspects. Scale bars = 5 mm. **b,** Electron microscopic axial image of the neurite bundles consisting of condensed neural fibers mainly filled with neurofilaments. Scale bar = 500 μm.
