# Supplementary material for: Novel artificial nerve transplantation of human iPSC-derived neurite bundles enhanced nerve regeneration after peripheral nerve injury
Source: Inflamm Regen. 2024 Feb 13;44:6. doi: 10.1186/s41232-024-00319-4 (PMC10863150; doi:10.1186/s41232-024-00319-4)
Supplement: Supplementary file 2 — Additional file 2: Figure S2. Images of ankle joint ROMunder anesthesia at 12 weeks. [file 41232_2024_319_MOESM2_ESM.pdf]

Supplementary Figure. 2

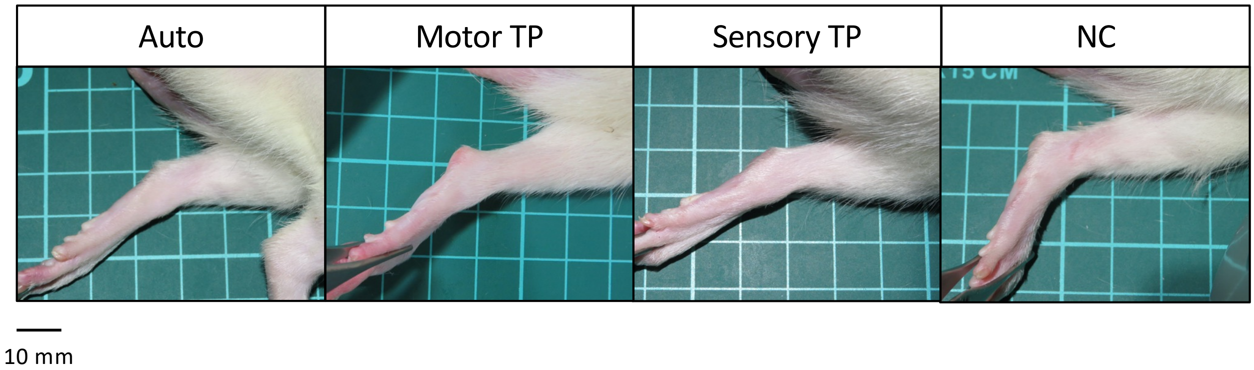

Supplementary Figure. 2

Images of ankle joint ROM under anesthesia at 12 weeks.

These images show ankle joint contracture in the NC group, Scale bar = 10 mm.
