# Supplementary material for: Novel artificial nerve transplantation of human iPSC-derived neurite bundles enhanced nerve regeneration after peripheral nerve injury
Source: Inflamm Regen. 2024 Feb 13;44:6. doi: 10.1186/s41232-024-00319-4 (PMC10863150; doi:10.1186/s41232-024-00319-4)
Supplement: Supplementary file 3 — Additional file 3: Figure S3. There was no significant difference between the transplantation group with 6 neurites’ bundles and that of 12 bundles in the recovery of motor and sensory functions. [file 41232_2024_319_MOESM3_ESM.pdf]

Supplementary Figure. 3

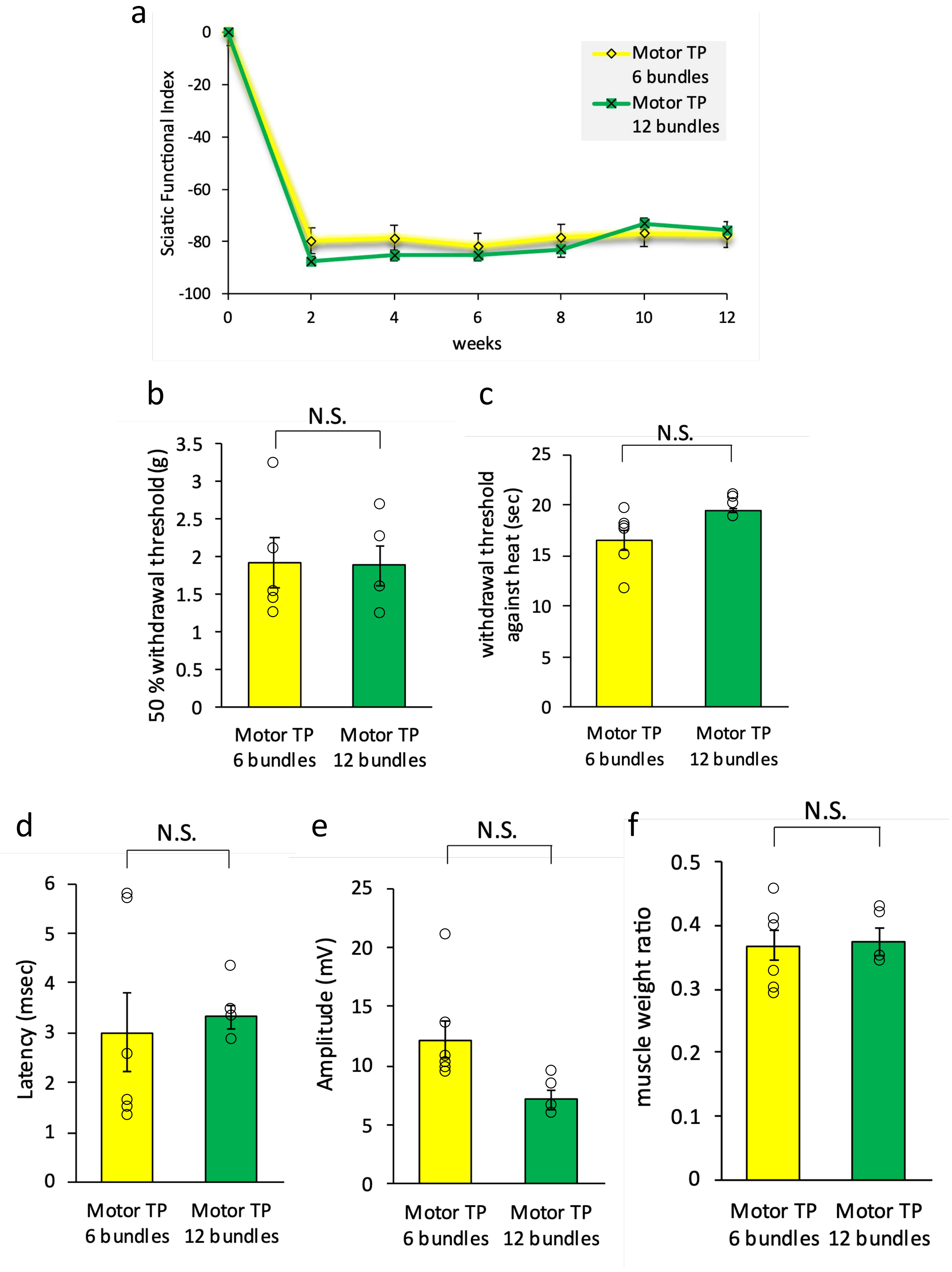

Supplementary Figure. 3

There was no significant difference between the transplantation group with 6 neurites' bundles and that of 12 bundles in the recovery of motor and sensory functions.

a, SFI over time up to 12 weeks after transplantation. **b-c,** Results from the von Frey monofilament test for touch sensation and from the Hargreaves plantar test for thermal sensation. **d-e,** Evaluation of latency and amplitude of compound muscle action potentials. **f,** Recovery of the reinnervated gastrocnemius muscle wet weight ratio with the injured side divided by the healthy side. (6 bundles: n = 6, 12 bundles: n = 4) N.S. = not significant. Data are represented as the mean ± SEM.
