# Supplementary material for: Novel artificial nerve transplantation of human iPSC-derived neurite bundles enhanced nerve regeneration after peripheral nerve injury
Source: Inflamm Regen. 2024 Feb 13;44:6. doi: 10.1186/s41232-024-00319-4 (PMC10863150; doi:10.1186/s41232-024-00319-4)
Supplement: Supplementary file 4 — Additional file 4: Figure S4. Limited functional and histological recovery in immunocompromised rats. [file 41232_2024_319_MOESM4_ESM.pdf]

Supplementary Figure. 4

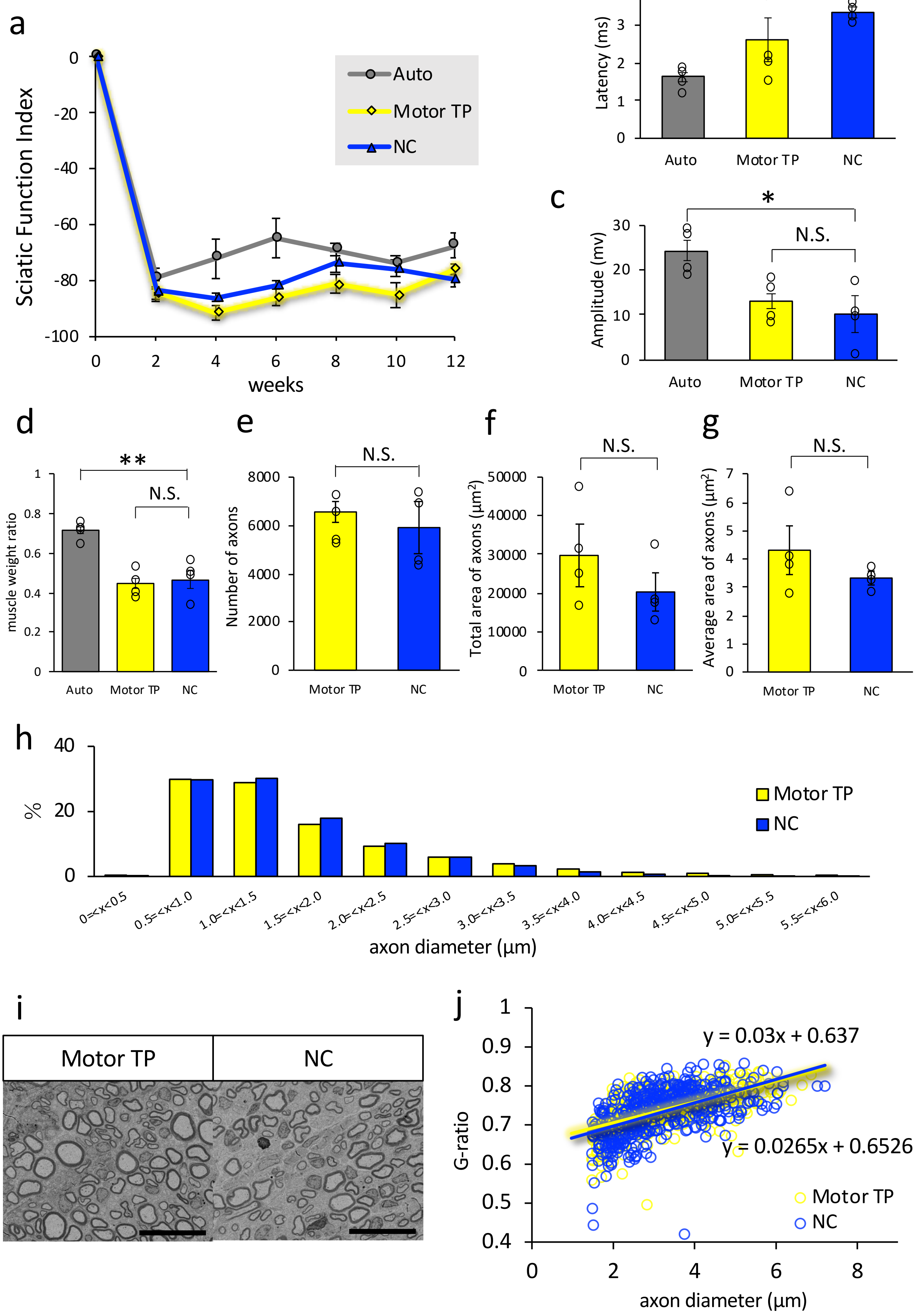

Supplementary Figure. 4

Limited functional and histological recovery in immunocompromised rats.

a, SFI over time up to 12 weeks after transplantation. **b-c,** Evaluation of latency and amplitude of compound muscle action potentials. **d,** Recovery of the reinnervated gastrocnemius muscle wet weight ratio with the injured side divided by the healthy side. **e-g,** Quantitative evaluation of regenerating axons. **h,** Distribution of axon diameter in the regenerating nerve. **i,** Representative electron microscopy images of central axonal area axial sections, Scale bars = 20 μm. **j,** Quantitative analysis of myelination with G-ratio calculations. (n = 4) $*p < 0.05$, $**p < 0.01$, N.S. = not significant. Data are represented as the mean ± SEM.
