# Supplementary material for: Novel artificial nerve transplantation of human iPSC-derived neurite bundles enhanced nerve regeneration after peripheral nerve injury
Source: Inflamm Regen. 2024 Feb 13;44:6. doi: 10.1186/s41232-024-00319-4 (PMC10863150; doi:10.1186/s41232-024-00319-4)
Supplement: Supplementary file 5 — Additional file 5: Figure S5. Bioluminescence live cell imaging experiments with IVIS. [file 41232_2024_319_MOESM5_ESM.pdf]

Supplementary Figure. 5

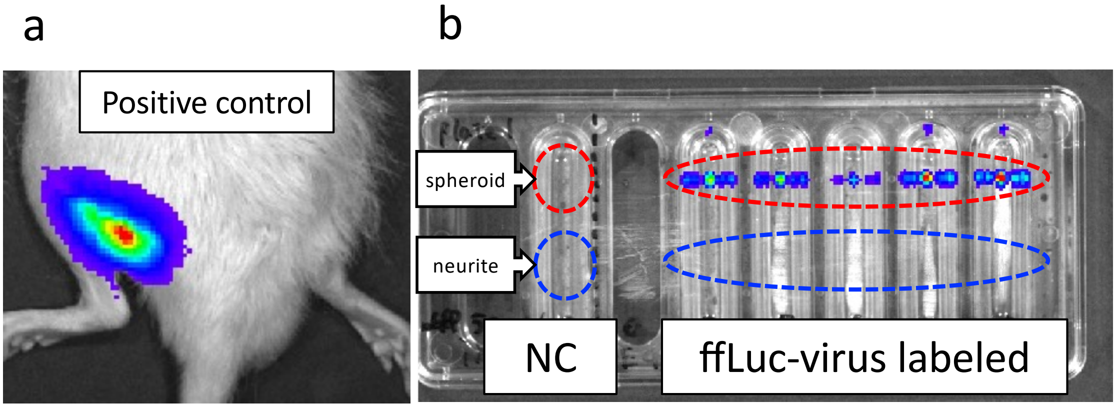

Supplementary Figure. 5

Bioluminescence live cell imaging experiments with IVIS

a, iPSC-derived neural crest-like cells (38) labeled with ffLuc lentivirus (20) were transplanted into the injured site of the left sciatic nerve. The ffLuc-expressing live cells were detected by IVIS.

b, Cultured nerve organoids labeled with ffLuc lentivirus in a microfluidic device were observed by IVIS. The bioluminescence signals were clearly detected from the live cells in spheroids but not from the neurite bundles.
